# A Case of Voltage‐Gated Calcium Channel and TG6 Antibody–Positive Cerebellar Ataxia

**DOI:** 10.1155/crnm/5563623

**Published:** 2026-04-25

**Authors:** Alberto Cifelli, Zain Butt, Eva Cifelli, Marios Hadjivassiliou

**Affiliations:** ^1^ Department of Neurology, Queen’s Hospital, London, UK, queens-hospital.com; ^2^ Faculty of Medicine and Dentistry, Queen Mary University of London, London, UK, qmul.ac.uk; ^3^ Queen’s College, University of Cambridge, Cambridge, UK, cam.ac.uk; ^4^ Department of Neurology, Sheffield Ataxia Centre, Royal Hallamshire Hospital, Sheffield, UK, nhs.uk

**Keywords:** antivoltage-gated calcium channel autoantibody, cerebellar ataxia, magnetic resonance imaging, magnetic resonance spectroscopy, Scale for the Assessment and Rating of Ataxia, TG6 autoantibody

## Abstract

We present the case of a lady in her early 20s who developed over a few weeks progressive appendicular and limb ataxia and dysarthria. She was found to have a high titer of voltage‐gated calcium channel antibodies (VGCCAs) and was started on immunosuppressive and immunomodulating therapy with no further worsening of her neurological status. Subsequent testing revealed that she was also TG6 antibody–positive, and a gluten‐free diet was added to her management, following which improvement in cerebellar N‐acetyl‐aspartate (NAA) concentration (an index of neuronal health/integrity) was noted on magnetic resonance spectroscopy (MRS), while her clinical picture remained static.

## 1. Introduction

Voltage‐gated calcium channel antibody (VGCCA)–related ataxia is a rare but treatable condition that should be considered in all cases where immune‐mediated cerebellar disease is suspected. Our patient was notable for the lack of tumor association, the presence of an asymptomatic neuromuscular junction disorder, the finding of gluten sensitivity, and the response to treatment with corticosteroids, intravenous immunoglobulins, mycophenolate, and a gluten‐free diet (GFD), which led to stabilization of the clinical and radiological picture, but did not prevent significant neurological sequelae.

## 2. Case Presentation

A lady in her early 20s was admitted to the neurology ward with a 3‐week history of dysarthria and difficulty with handwriting, which was followed within a week by unsteadiness of gait. Just before hospitalization, she had noticed that her left hand was clumsy as well. There was no recent history of vaccinations, infection, or ongoing constitutional symptoms. She had no past or family medical history of note, was on no medications, and had never smoked.

On examination, there was mild gait ataxia, with a strained, staccato speech. Subtle horizontal nystagmus was evoked by right lateral gaze, and saccades were hypometric in all directions. Truncal, symmetrical upper and lower limb ataxia of moderate degree was elicited, and the arms were symmetrically dysdiadochokinetic.

Magnetic resonance imaging (MRI) of the brain and spine with contrast did not show any significant abnormalities. Computed tomography (CT) of the chest/abdomen/pelvis with contrast was reported to reveal a benign right ovarian cyst. The patient had nerve conduction studies, including repetitive nerve stimulation of the abductor pollicis brevis (APB) and trapezius, and single fiber EMG (SFEMG) of the orbicularis oculi muscle. They showed normal amplitude of the compound muscle action potentials (CMAPs) from all motor nerves tested (median, ulnar, common peroneal, and tibial), significant CMAP amplitude decrement on slow (2–5 Hz) repetitive nerve stimulation of APB and trapezius but no significant change post‐exercise. SFEMG of the orbicularis oculi revealed increased jitter and blocking. This was felt to be in keeping with a predominantly postsynaptic neuromuscular junction disorder. Whole‐body positron emission tomography–CT with fluorodeoxyglucose demonstrated asymmetrical uptake in the palatine tonsils (higher on the right) and mild uptake in the thymus; subsequent MRI of the soft tissues of the neck and ENT clinical review only highlighted a physiological disparity in tonsillar size and a repeat chest CT with contrast excluded thymic pathology. FBC, coagulation screen, U&E, LFT, calcium, phosphate, SARS‐CoV‐2 throat swab, B12, syphilis and HIV serology, CRP, CK, venous lactate, caeruloplasmin, copper, vitamin E, alpha‐fetoprotein, TPO and connective tissue disease antibody screen, ANCA, TTG IgA, anti‐GM1, anti‐GQ1b, Hu/Ri/Yo/Ma2/Tr, glutamic acid decarboxylase (GAD), islet cell, AMPA‐1 and 2, GABAb, acetylcholine receptor and Musk antibodies, and peripheral toxoplasma serology were normal or negative; immunoglobulin IgG were raised at 18.05, while IgA and IgM concentration was within normal limits; serum folate was low at 4.0 (normal range: 3.9–26.8 mcg/L). Serum voltage–gated calcium channel antibodies (VGCCA) were positive at 2191 pmol/L (normal range: 0–45). She had a lumbar puncture: the opening pressure was 28.5 cmH2O. The CSF was clear and colorless, and its constituents are as follows: protein 0.32, lactate 1.49, glucose 3.6 (contemporary serum levels were not measured), 4 lymphocytes, and 417 RBC. CSF microscopy, culture, enterovirus, HSV 1 and 2, and VZV PCR were negative; cytology did not reveal any malignant cells and there were oligoclonal bands in the CSF but not in serum. CSF anti‐GAD antibodies were also negative.

The patient was given 1 g of intravenous methylprednisolone a day for 3 consecutive days, followed by oral prednisolone 40 mg once a day for 1 month. At the end of this course of therapy, as there was no clinical improvement, intravenous immunoglobulins (2 g/kg over 5 days) were administered.

Six months after presentation, and whilst still on steroids, the patient was reviewed at the Sheffield Ataxia Center, and additional serological testing showed positive TG6 antibodies, suggestive of gluten sensitivity with a propensity to neurological manifestations. She also underwent her first MR spectroscopy (MRS) of the cerebellum. N‐Acetyl‐aspartate (NAA) to creatine (CR) area ratio (NAA/CR) in the cerebellar vermis was significantly low at 0.61. The value from the right hemisphere was also significantly low at 0.69 (both values should be well above 1 in healthy individuals). The patient was advised about adopting a strict GFD, and mycophenolate 500 mg bd was introduced, which was increased to 1 g bd 1 month later, while a slow oral steroid taper was commenced. Repeat MRS scanning 5 months after starting mycophenolate and on a GFD showed significant improvement with the NAA/Cr ratios in the vermis and hemisphere increasing to 0.86 and 0.76, respectively.

The patient then decided, of her own accord, to reduce the mycophenolate to 500 mg bd due to nausea and fatigue, and, within 2 weeks, on routine blood monitoring she was noted to be neutropenic, which led to a further dose reduction to 500 mg od. As the neutrophil count declined further, mycophenolate was stopped, but was restarted 2 months later when the neutrophil count normalized, returning over several months to 1 g bd. She remained on this regimen until, at 29 months from her presentation, she developed a transaminitis (with ALT at 373 U/L [normal range < 33 U/L]) whose cause could not be established by a hepatologist, and mycophenolate was discontinued. She continued on a strict GFD with complete elimination of the TG6 antibodies.

The patient has now been under clinical follow‐up for a total of 5 years, during which her Scale for the Assessment and Rating of Ataxia (SARA) score has stabilized around 6 (from a baseline of 7.5–8), and no evidence of neoplastic disease has emerged. The most recent MRS showed similar readings to the last one.

## 3. Discussion

Pure cerebellar ataxia secondary to anti‐VGCCA is a rare entity, having been described in around 90 cases to date.

In 1973, Satoyoshi et al. [1] first clearly characterized a patient with small cell lung cancer (SCLC), who had Lambert–Eaton myasthenic syndrome (LEMS) and pathologically confirmed paraneoplastic cerebellar degeneration (PCD); however, it seems plausible that two of the three cases reported in detail in a series of 19 patients with “subacute cerebellar degeneration associated with neoplasms” by Brian and Wilkinson in 1965 [2] also had LEMS. These and subsequent similar observations [3, 4] led to the awareness that cerebellar ataxia co‐occurred with LEMS more frequently than expected by chance.As VGCCA are almost invariably present in LEMS, and calcium channel mutations were proved to determine genetic forms of ataxia [5], their pathogenetic role in the etiology of PCD was posited. Over time, it has become apparent that a proportion of cases of autoimmune cerebellar ataxia can also manifest themselves in the absence of neoplasia (which is usually SCLC, small cell prostate cancer or Hodgkin’s lymphoma [6], clinical evidence of LEMS, and other antibody markers of paraneoplastic disease [7]). It is estimated that < 10% of LEMS patients with VGCCA develop ataxia [8], but peripheral neurological manifestations may obscure cerebellar signs and lead to an underestimate [9].

There is persuasive evidence supporting pathogenetic roles for VGCCA in cerebellar ataxia. Among the many types of neuronal voltage–gated calcium channels in existence, it is the P/Q ones that are reputed to be causal for both LEMs and cerebellar ataxia: they are present at the presynaptic motor nerve terminal but were first identified on the surface of cerebellar Purkinje cells [10].

In 2003, Fukuda et al. [11], using human autopsy cerebellar tissues from three PCD‐LEMS patients, found that the number of P/Q type VGCC was found to be reduced by 80%, particularly in the molecular layer, and autologous IgG to be bound to the VGCC channels remained. In another study [7], VGCCA from a patient with autoimmune cerebellar degeneration and LEMS bound to Purkinje cells in rat cerebellar tissue: this was followed by scattered Purkinje cell death. Importantly, VGCCA have also been shown to have a functional effect on cerebella tissue. A 2008 study [12] used a polyclonal peptide antibody against a major immunogenic region in P/Q‐type VGCCs (the extracellular Domain‐III S5–S6 loop), demonstrating that it was sufficient to inhibit VGCC function in neuronal and recombinant VGCCs, alter cerebellar synaptic transmission, and confer the phenotype of cerebellar ataxia in mice. Martin‐Garcia et al. [13] evaluated in mice the effect of intrathecal injection of IgG purified from the serum of a patient with both PCD and LEMS, and from another patient with isolated LEMS: mice injected with PCD/LEMS IgG developed reversible ataxia compared to those injected with LEMS or control IgG; when injected with IgG from the patient with pure LEMS, ataxia was not induced, suggesting distinct epitope specificity.

Our patient’s nonparaneoplastic cerebellar ataxia syndrome is notable for the neurophysiological finding of a neuromuscular junction disorder at presentation in the absence of clinical manifestations. Her ataxia stabilized after treatment with corticosteroids, intravenous immunoglobulins, mycophenolate, and GFD, but she remains disabled by significant dysarthria, appendicular ataxia, and unsteadiness of gait and needs to use a walking stick. Cerebellar MRI and MRS findings demonstrated initial improvement and then a plateau, which was reflected clinically by the SARA score. Whilst data from the literature [7, 14] suggest that therapeutic outcomes in this condition are often disappointing in spite of early immunotherapy, in our patient, there has been no evidence of clinical or radiological worsening after initial improvement. A possible explanation for this is that the patient was also found to be gluten‐sensitive: as a consequence, a strict GFD was instituted, which may have helped in achieving the results observed. The possible relative contribution of the GFD merits further consideration. Ataxia related to VGCCA is extremely rare; however, gluten ataxia is one of the most common immune ataxias. Coexistence of two autoimmune diseases is not an uncommon phenomenon. It is therefore difficult to tease out the relative contribution of the GFD in this patients’ recovery/stabilization because she was started on mycophenolate at the same time as starting the GFD. We did not wish to risk treating her with just GFD, given the aggressive and progressive nature of the initial presentation and the presence of VGCCA.

Immune ataxias form a substantial part of the progressive ataxias and merit diagnostic consideration as they are potentially treatable. A number of autoantibodies have been reported in association with cerebellar ataxia, and VGCCA should be included in laboratory screening, including in the setting of idiopathic late‐onset syndromes, where its prevalence is significant (11.9% according to Burk et al. [15]). Important clues suggestive of immune ataxia include the rapidity of onset (as was the case here), the rapid progression, and the primarily vermian involvement on imaging.

## Funding

This study received open access publication funding from a transitional agreement between Wiley Publishing and Queen Mary University of London.

## Disclosure

The manuscript was presented at the 2026 Annual Meeting | P9‐Poster Session 09.

## Consent

All the patients allowed personal data processing, and informed consent was obtained from all individual participants included in the study.

## Conflicts of Interest

The authors declare no conflicts of interest.

## Data Availability

The data that support the findings of the study are available upon reasonable request from the authors.
